# The path from a volunteer initiative to an established institution: evaluating 15 years of the development and contribution of the Lighthouse trust to the Malawian HIV response

**DOI:** 10.1186/s12913-017-2466-y

**Published:** 2017-08-09

**Authors:** Sam Phiri, Florian Neuhann, Nicola Glaser, Thomas Gass, Thom Chaweza, Hannock Tweya, Lighthouse Group

**Affiliations:** 1 Lighthouse Trust, Lilongwe, Malawi; 2 Department of Medicine, University of North Carolina School of Medicine, Chapel Hill, USA; 3 Department of Public Health, University of Malawi, College of Medicine, School of Public Health and Family Medicine, Lilongwe, Malawi; 4 Institute of Public Health, University of Heidelberg, Heidelberg, Germany; 5 Vivantes Klinikum Neukölln, Kinder- und Jugendmedizin – Perinatalzentrum, Berlin, Germany; 6 Swiss Red Cross, Bern, Switzerland; 7 London School of Hygiene and Tropical Medicine, London, UK

**Keywords:** Malawi, HIV, Aids, Public health initiative, Health service delivery, Resource limited setting

## Abstract

**Background:**

The HIV epidemic has triggered the development of new health institutions with a special focus on HIV care. The role of these relatively new institutions within the health systems of low-income countries like Malawi is not clearly determined. We evaluate and describe the development of one example, the Lighthouse Trust (Lighthouse), over a period of 15 years (2000–2015).

**Methods:**

Data from multiple sources, including a document review, participatory observation and interviews were analysed, triangulated and synthesized. The institution’s development, function, cooperation, financing, research and training were analysed using institutional administrative documents, annual reviews, project reports. For the assessment of the research activities, all publications that the Lighthouse contributed to were retrieved and categorized. Participatory observation and interviews with key Lighthouse staff members and external stakeholders were conducted.

**Results:**

Established in 1997 as a volunteer initiative for home-based care, the Lighthouse has developed considerably. Major steps include being registered as a trust, moving into their own buildings, expanding clinical services, becoming a centre for clinical service, training and research working with close to 300 employees. As an independent legal entity, Lighthouse Trust works in close cooperation with Malawian public health services and plays an important role in the government’s HIV programme. Funding comes from various sources with a lion’s share from the US Centers for Disease Control and Prevention. Throughout 2015, the Lighthouse performed 58,210 HIV testing and counselling encounters and by year’s end, 28,302 patients were alive and on ART. From 2000 to 2015 Lighthouse staff contributed to 94 peer-reviewed publications.

**Conclusion:**

Novel institutions like the Lighthouse have been developed in the response to HIV. The Lighthouse has demonstrated its capacity to deliver health services and contributed significantly to the current level of success in addressing the disease. However, this kind of institution’s position in local health care systems is still developing. The Lighthouse will need to continue to work on well-planned strategies that consider the changing landscape of health needs, health care provision and financing. Independent institutions like the Lighthouse can contribute to the development of health systems in countries like Malawi that improve health care responsiveness and quality for the entire population.

**Electronic supplementary material:**

The online version of this article (doi:10.1186/s12913-017-2466-y) contains supplementary material, which is available to authorized users.

## Background

Since AIDS was first described in 1981, HIV has become one of the deadliest diseases in the world. Approximately 37 million people currently live with HIV (PLHIV) and 39 million having died [1, 2]. Sub-Saharan Africa (SSA) carries 70% of the global burden of HIV infection [3]. In March 2015, there were 15 million people accessing antiretroviral therapy (ART), which corresponded to approximately 40% of all PLHIV worldwide. The significant AIDS epidemic and attendant consequences have made new solutions necessary.

The HIV epidemic has stimulated community-based disease initiatives and fostered the establishment of many organisations and institutions to address the effects of HIV and AIDS. This in turn has resulted in a number of initiatives. One of the most effective and well-known initiatives in the field is TASO (The AIDS Support Organisation), which was founded in 1987 in Uganda as a support group. TASO has now evolved into a non-governmental organization (NGO), operating across Uganda. TASO delivers services, develops capacity and encourages research in the field of HIV and AIDS [4].

In Malawi, similar initiatives for community support, orphan care, organisations of PLHIV, counselling and testing centres, and treatment have been established. One of the most renowned organisations is the Lighthouse Trust, which is situated in the capital, Lilongwe.

Malawi is situated in the southeastern part of the African continent and has been greatly affected by HIV. Ten per cent [5] of the 17 million inhabitants are estimated to be living with HIV. Although Malawi is one of the poorest countries in Africa, with more than 60% of people living below the poverty line [6], it has reached an ART coverage of more than 50% [7]. At the end of September 2015, 585,660 patients were alive and on ART, reflecting that 59% of the estimated 1 million PLHIV were on ART. Estimated ART coverage among people in need for treatment was 50% (50,533 / 101,000) for children (<15 years) and 68% (531,990 / 779,000) for adults.

Malawi’s health system and services are dominated by the public/governmental sector. Other important actors are faith-based organizations, predominantly the Christian Health Organization of Malawi (CHAM), NGOs and private clinics. The country has a high disease burden of communicable and non-communicable diseases as well as maternal and child health problems [8]. Despite this significant need for health facilities and trained personnel, in 2008, there were only 257 physicians in Malawi, which is about one per 50,000 inhabitants [9]. Two thirds (68.3%) of the total expenditure on health comes through external resources [10].

The Lighthouse Trust (Lighthouse) represents a new type of institution in the Malawian health system. The Lighthouse was founded by governmental health workers and was registered as a Public Trust incorporated under the Malawi Trustees Act (1962) in 2001. It is governed by a board of trustees. The Lighthouse can be characterized as a government-partner institution, not faith-based or for-profit, but with a clear focus on integrated HIV prevention, treatment, and care. The Lighthouse is closely linked to public service, yet initially, it had a new or previously undefined position in the health care system.

In this case study, we describe the Lighthouse’s organizational model, and reflect on the impact of this comparatively new institution on Malawi’s health system. The motivation to conduct this case study was to achieve a better understanding of what Lighthouse does, how it works and is organized and what it has achieved in service provision, training and research over a period of 15 years (2000–2015). This period is characterized by multiple changes in the national HIV epidemic and the response to it- in particular with regards to the massive expansion in access to treatment. Changes also occurred in national politics, donor policies and funding. This case study can serve as an exemplar for other low-income countries facing similar challenges in addressing the continuing HIV epidemic.

## Methods

This is a case study of an institution and programme (the Lighthouse) developed to address HIV and AIDS and situated in the capital of Malawi, Lilongwe. An organisational and policy analysis (OPA) of the Lighthouse was conducted in 2011 and thus, the decision was made by the authors to perform this case study to examine Lighthouse’s development from 2000 to 2015. This process was completed in January 2016. The guiding themes for the case study were:

- History and organisational structure of the Lighthouse
- Function and funding within the Malawian health system
- Provision and proliferation of clinical services, training and research.

This case study triangulates, synthesizes and interprets the following data sources: document review, structured and informal participatory observations and interviews with Lighthouse staff members and external stakeholder representatives [11].

To describe the Lighthouse’s organizational structure, practices, interventions, achievements and challenges, relevant documents from 2000 to 2015 were retrieved and analysed (Table 1) (see Additional file 1). Performance data from annual reports (beginning in 2002) to the Board of Trustees and the National HIV Programme and funders were also reviewed. Organisational information was obtained from the Lighthouse’s management manual and the authors’ personal work experiences.

**Table 1:** Major documents analysed for the case study in chronological order

Type of source	Name of document	Purpose	Methods	Author	Year
Case Study	The Lighthouse: A centre for comprehensive HIV/AIDS treatment and care in Malawi	Description of antiretroviral therapy perspectives and practices	Observations Interviews Document review	Phiri et al. (internal)	2004
Report	Institutional Review	Describe the current status and inform strategic development	Observations Interviews Document review	Salephera Consulting (external)	2010
Organisational and policy analysis (OPA) report	Innovation, operational research and scaling-up in HIV care: Lessons learnt from the Lighthouse, Malawi	Performed in partial fulfilment of a doctorate in public health	Observations Interviews Document review	Thomas Gass (visiting temporary LH- member)	2011
Report	Formative research to inform clinic efficiency: Qualitative assessment of patients’ opinions and perceptions of Lighthouse Trust clinic services	Patients’ perspectives on Lighthouse services	Qualitative research, Focus group discussions and in-depth interviews	REACH Trust (external)	2012

The OPA (performed by co-author TG) applied the following additional methods: open-ended face-to-face interviews with 30 respondents, of which 20 were Lighthouse staff members and ten were external stakeholders—representatives from partner institutions. Lighthouse staff interviews included the then seven members of the management team (the Executive Director, the Manager, and Coordinators of the Departments for Training, Clinic, Home-Based Care, HIV Testing and Counselling and Monitoring & Evaluation [M&E]), eight clinicians and nurses and five M&E and administrative personnel. Selected staff members had to be employed for at least 2 years at the Lighthouse and involved in operational research and innovations.

External interviewees were chosen based on close cooperation with the Lighthouse and represented the following institutions: Ministry of Health (MoH), National AIDS Commission, REACH Trust (an independent Malawian social science research institution), Baobab Health Trust (an independent Malawian health information institution), the Lilongwe Project of University of North Carolina (a major academic partner for service and research) and two leading teaching hospitals, Kamuzu Central Hospital, Lilongwe and Queen Elisabeth Central Hospital, Blanytyre. Interviews focussed on new or piloted interventions that led to changes in practice, and thus informed or were accepted into Malawi’s national HIV programme. During the interviews, notes were handwritten and then immediately transcribed into a computer.

Participatory observation during the OPA analysis took place with members of the M&E Department and focussed on two operational research questions: 1) the total time patients spend in the clinic from registration to drug dispensing and 2) the capacity of trained Lighthouse volunteers to visit ART clients at home for adherence and psychosocial support. (Results of the operations research served for internal use and are not presented in the case study).

To assess the research activities conducted by or together with the Lighthouse, we searched Pubmed for authors who were Lighthouse staff members and Lighthouse as an affiliation or study site to describe the organisation’s contribution to HIV care delivery, capacity building, and operations research. Pubmed was accessed for publications dated January 1, 2000 to December 31, 2015 and last accessed on January 6, 2017.

### Ethics

The case study authors used available internal documents for analysis. The protocol for the structured participatory observation and the staff interviews received Institutional Review Board clearance by the London School of Hygiene and Tropical Medicine (Application No 5920 March 2011) and through the National Health and Science Research Committee Malawi (NHSRC Protocol # 898, June 2011) All staff members interviewed were asked for and signed a consent form. Clients/patients were not directly involved in interviews or observations. All staff involved were bound to patient confidentiality and data was protected by attendant professional discretion.

## Results

### Description of setting and development of the institution

#### History

The Lighthouse started in 1997, when staff of the medical ward and other departments of Kamuzu Central Hospital (KCH), a tertiary hospital in Malawi’s central region, felt the urgent need to help patients and community members in the face of the HIV epidemic by providing HIV and AIDS care and support. Staff members who were off-duty worked with community volunteers to begin home visits and offered HIV counselling, testing and treatment for opportunistic infections. Over time, the process became more organised and a major step in the institutional development process was the founding of the Lighthouse Trust (2001) [12] followed by housing in a newly rehabilitated building on the KCH campus in 2002 that was funded with support from the European Union [13]. The aforementioned process was driven by Malawian health professionals and supported by international partners such as the WHO, the ProTest Project (NORAD), the Centre for International Migration and Development, (CIM, Germany) and the University of North Carolina Lilongwe Project (UNC, USA) [2, 14].

Antiretroviral treatment started slowly in 2000 as a fee-for-service program financed by the Malawian government in a revolving fund, i.e. patient payments were used to buy new drugs. ART cost sharing continued until in June 2004 when ART became free-of-charge supported by the Global Fund.

The history of Lighthouse over this period is further reflected by the process of developing mission statements. Initially in 2003, the Lighthouse Mission Statement expressed that “The Lighthouse Trust exists to fight against AIDS in Malawi by providing a continuum of quality care and support and by working to build capacity in the health sector”. In 2014, Lighthouse acknowledged that instead of “fighting” the mission statement should reflect the current approaches and that HIV had turned into a treatable chronic disease also in Malawi, hence revised the mission statement to be “The Lighthouse Trust contributes to Malawi’s national response to HIV as a model in providing a continuum of high quality care and building capacity in the health sector”.

**Fig. 1:**
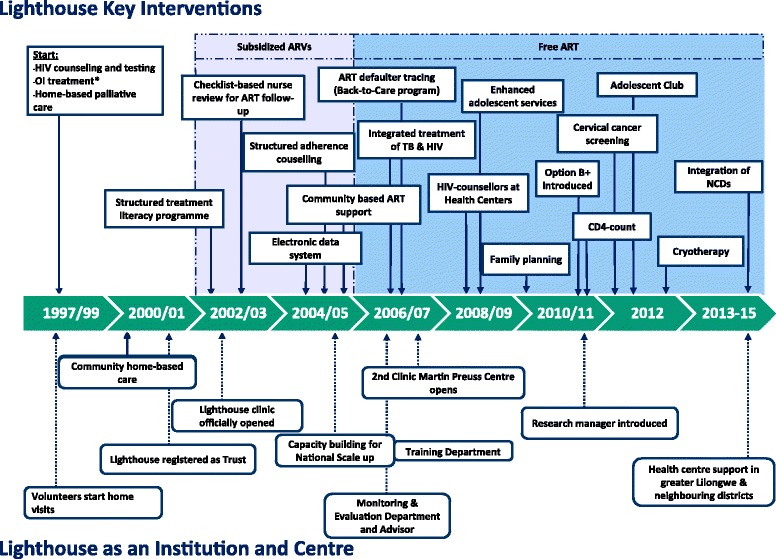
Timeline for key interventions and steps in institutional development 1997–2015. Abbreviations: *OI* opportunistic infection, *ART* antiretroviral treatment, *TB* tuberculosis, *CD4* CD4+ T helper cells, *NCDs* non-communicable diseases

The further timeline of the development of the activities are depicted in Fig. 1.

#### Organisational and administrative structures

Lighthouse evolved from a group of volunteers in the late 1990s to a fully institutionalized organisation with close to 300 employees. Administrative structures have been adjusted regularly to reflect institutional evolution and growth [13].

**Fig. 2:**
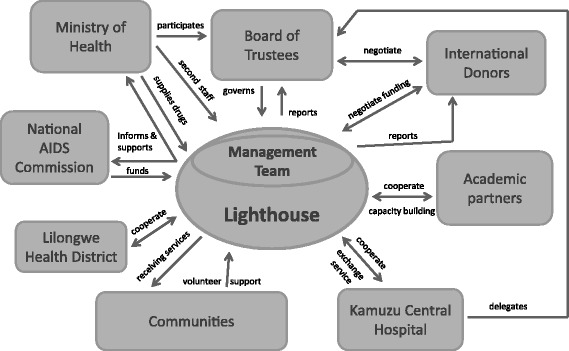
Organisational Structure/Institutional frame. Unidirectional *arrows*: symbolize main direction of interaction. Bidirectional *arrows*: symbolize mutual exchange, negotiations

There is a good relationship between the Ministry of Health (MoH), which participates in the Lighthouse Board, and the Lighthouse, which gives direct information back to the Ministry. The Lighthouse also has a long history of cooperation with academic partners on a national level with the Colleges of Medicine, Nursing and Health Sciences among others and on an international level with the Universities of North Carolina, Liverpool, London, Cologne and Heidelberg. This enables all sides to cooperate, exchange services and strengthen capacity building. Lighthouse is also strongly interlinked with KCH, Bwaila Hospital and the Lilongwe District Health Office and communities that offer volunteer support and receive health care services [13, 15]. The multiple relationships of the Lighthouse are shown in Fig. 2.

**Fig. 3:**
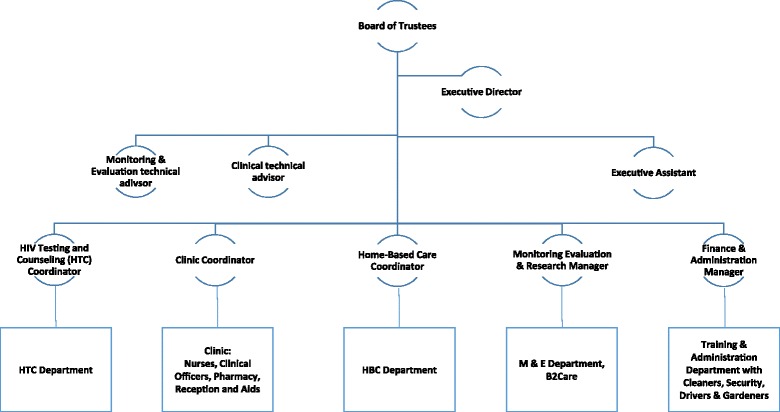
Simplified organigram of the Lighthouse structures. The management organigram changed in 2016 and now includes a Financial and Administration Director, a Monitoring and Research Director and a Medical Director

The Lighthouse as a Trust is governed by a Board of Trustees, which appoints the Executive Management including the Executive Director. A comprehensive management manual describes and regulates all operations at the Lighthouse and includes sections on finance, procurement, administration, human resource recruitment and management [16] according to international and national standards. Final decision making lies with the Executive Director who plans and operates the Lighthouse in close cooperation with the Executive Management (Finance and Administration Director, Monitoring, Evaluation and Research Director, Monitoring, Evaluation and Research Technical Advisor, and Clinical Advisor) and the management team (see Figs. 2 and 3).

Annual retreats by the management team and occasionally by other departments are another Lighthouse feature as well as daily staff meetings and weekly departmental group meetings. These meetings provide for internal communication and development of strategic plans or short-term operational adjustments.

A hallmark of Lighthouse’s structure is being rooted in the communities which it serves and operates within. A number of employees, including PLHIV, started as Lighthouse volunteers and were recruited as staff when opportunities arose. Activities with community volunteers remain a regular event and provide opportunities for feedback [12]. A qualitative study conducted by an independent research group with patient focus-group discussions at the Lighthouse provided critical feedback. The group were able to point out critical aspects of care, e.g. the attitude of some Lighthouse employees towards patients seeking care outside their scheduled appointments [17].

#### Funding

Financially, the Lighthouse draws from national and international sources including the Government of Malawi through the MoH and National AIDS Commission with pooled funding including the Global Fund to fight AIDS, tuberculosis and malaria; United Sates of America (The U.S. President’s Emergency Plan for AIDS Relief (PEPFAR) through Centers for Disease Control and Prevention,CDC); Catholic AID for Overseas Development (CAFOD); Irish Aid through the Rose Project; International Epidemiologic Databases for Evaluating AIDS – Southern Africa; World Health Organisation; University of North Carolina; Deutsche Gesellschaft für Internationale Zusammenarbeit (GIZ incl. (CIM, ESTHER)) and independent charitable foundations. During the early years, a number of employees were funded by the Government of Malawi (GoM); whereas today, the GoM supports Lighthouse with drugs and other supplies. The lion’s-share of funds for staff and operations comes through the aforementioned agencies particularly the CDC, and is performance-based and oriented to specific activities. For a few years now, the Lighthouse has been acting as a CDC local implementing partner, and creates most of the budgets with major implications on the operations. Strategically, however, the Lighthouse has always sought to diversify their funding sources to reduce dependency on a single donor.

#### Service provision

Lighthouse’s services are based on a three-pronged technical approach:

1. Service delivery to scale via expanded, high-quality HIV and AIDS treatment care and support services provided through the Lighthouse, and
2. Service delivery as a model intervention and operations research with innovative approaches for HIV treatment, care and support developed, piloted, evaluated and disseminated in Malawi, and
3. Capacity building to strengthen capacity for HIV and AIDS treatment, care and support in Malawi.

##### Clinical care

The Lighthouse started supplying subsidized antiretroviral medication in 2002 and was the first clinic in Malawi to supply ART free of charge in June 2004 since it had a well-established clinic. It became a certified centre in 2005 for the national scale-up of ART and conducting training courses and clinical attachments. In 2006, the second clinic, the Martin Preuss Centre, was opened at Bwaila Hospital and a Monitoring and Evaluation Department was established and supported by a technical advisor to maintain quality.

In order to address the large number of patients, many innovations have been developed and integrated in the provision of care. Beginning in 2002, group-based pre-treatment structured adherence counselling and treatment literacy programs were introduced as key elements to ART provision [11].

A checklist-based review form was developed to enable a standardized ART follow-up by nurses as early as 2003 [11]. The transition to nurses supplying ART based on a checklist, instead of only clinicians, enabled the Lighthouse to more efficiently address the increasing numbers of HIV patients long before task shifting became a recommended strategy [18]. The checklist controls for signs of opportunistic infections and severe side effects and enables the nurses to refer patients with these symptoms to clinical officers. This innovation was adopted by the MoH and integrated into the national ART guidelines in 2006, which enabled a nationwide transition [19].

At the Lighthouse, the checklist was integrated into an electronic medical record system (EMRS) that is now operated via touchscreen monitors from all points of the clinic and connected to a central server. After this system was introduced at the Lighthouse, it was then transferred to over 70 other larger HIV treatment centres in Malawi.

Barcode labels are printed and placed into the patients’ personal health passports. This enables all Lighthouse workers to instantly recall patients’ healthcare information via barcode scanning [15].

Community-based ART support and ART defaulter tracing, called “Back to care”, was gradually integrated into the regular healthcare process. When a patient misses an appointment by more than three weeks, the system notifies the Back-to-care team, which then traces the patient via phone or home visit [20]. One of the most specific Lighthouse interventions is the “Ndife Amodzi” programme, which is a community-based intervention carried out by volunteers to psychologically support patients and strengthen ART adherence.

The Lighthouse supported the establishment of the first paediatric ART clinic at KCH. Many processes have been piloted and evaluated there. Currently, the Lighthouse has on-going cooperation for paediatric HIV care with other providers on campus such as the Baylor Centre of Excellence and the paediatric Department of KCH. Many children from the initial Lighthouse paediatric cohort are being followed into adolescence and new models of care for this age group including a youth club have been established [21].

In 2006, services for tuberculosis and HIV were integrated by building a second Lighthouse clinic. Targeted protocols, trainings and monitoring tools were developed and evaluated and became the standard of care for Malawi when the MoH launched the national rollout of ART in tuberculosis (TB) clinics in 2010 [22, 23].

More recent service developments have been the introduction of point-of-care CD4 counts (2011), and family planning and cervical cancer screening (2012) and these have become the standard of care.

##### HIV testing and counselling

To enable patients living in the more rural parts of Lilongwe District to receive adequate health care, Lighthouse started training counsellors in peripheral health centres. The task of HIV counselling and testing has been shifted to non-medical personnel after receiving special training. This has enabled the Lighthouse clinics to spare nurses for ART follow-up despite having high numbers of clients for HIV testing and counselling (HTC). The Lighthouse supports public HTC services in 10 health centres in Lilongwe and 51 facilities in the Dedza, Mchini and Ntcheu districts.

##### Community health care services

In the early phase of volunteerism, home-based care was one element of the Lighthouse’s service provision. Nurses provided care, but also trained family members and community volunteers to support AIDS patients by providing basic nursing, nutrition support, pain relief, and bereavement visits after a patient had died. This initiative contributed to the start of palliative care in Malawi [12, 13]. The development of the total number of clients and patients as primary beneficiaries are shown in Table 2. The Lighthouse‘s operations are characterized by the combination of health care services, monitoring and evaluation, research and capacity building via training and skill sharing. In this way, it has reached more than 28,000 patients, who are HIV-positive and receiving ART via the Lighthouse. In particular, the data for HTC services show the influence of concurrent programmes that provided funding for additional counsellors for the testing services. Depending on counsellor and test kit availability, the numbers of HTC services varied widely over the course of 15 years and do not show a continuous increase (see Table 2). In 2012, a new category of “pre–ART” was introduced into the Malawian national HIV programme. Unfortunately, a reclassification of all Lighthouse patients was not feasible.

**Table 2:** Synopsis of Service Development at Lighthouse

Service	1997-2000^a^	2001	2003	2005	2007	2009	2011	2012	2013	2014	2015
VCT/HTC encounters	^b^	^b^	6909	15,039	26,240	40,939	41,706	39,799	41,567	46,944	58,210
CHBC clients	^b^	^b^	557	1498	2211	2642	3051	3246	3406	3549	3681
Non-ART Patients^d^	18	57	619	1206	1670	2087	1977	^d^	11,258	14,727	17,188
ART ever started	7	56	1969	5735	10,142	18,740	30,228	^d^	43,671	50,171	56,611
ART alive & continuing	^b^	^b^	836	2620	4570	11,231	17,412	^d^	22,419	25,053	28,302
TB/HIV patients	na	na	na	^b^	761	5821	10,726	10,420	11,742	12,714	13,730
HTC Health Centre	na	na	na	na	na	109,891	91,565	55,462	65,852	123,718	88,852^c^
Family Planning Clinic Clients	na	na	na	na	na	na	1198	2180	2687	3529	4247
Adolescent Care Members	na	na	na	na	na	25	25	37	64	300	340

Figures represent the total number of patients (cumulative) at the end of the respective year

Table 2 Lighthouse Services

*VCT/ HTC* Voluntary counselling and testing/HIV testing and counselling, *CHBC* Community home based care

^a^Estimates, no systematic documentation

^b^Data not available

^c^From September to December 2015, there were no counsellors in the Health Centres

^d^These figures are valid for patients who were not-yet-eligible for ART according to Malawian National Guidelines until 2011 and from 2012 onwards for “pre-ART patients”

#### Capacity-building training

The Lighthouse has a vivid training and skill-sharing culture. Within its institutional culture with daily morning meetings when daily work is discussed and regular (weekly) departmental meetings, the Lighthouse staff has developed a critical and self-reflecting attitude and fluent communication channels. The meeting participants are encouraged to critically reflect on their practices and report newly learned skills. There are frequent weekend or lunchtime trainings and workshops and the whole staff is encouraged to participate. The Lighthouse sends staff to external trainings for continuous professional development and develops staff capacities through their own trainings [13]. This has resulted in personal development to formal academic levels ranging from a diploma through BSc, Masters and PhD. Trainings are conducted with external partners such as HIV programme /MoH, and since 2012, the ESTHER partnership with the university clinics of Cologne and Heidelberg [24]. Lighthouse staff is key in the trainings conducted by the MOH /HIV programme and contributes significantly to national capacity building. Since Lilongwe has become a campus of the College of Medicine, Lighthouse is involved in the education of third-year medical students [15].

#### Research at lighthouse

From the early times of ART delivery, Lighthouse operations/implementation research has been a feature of the Lighthouse documented by a considerable number of publications and presentations at international conferences and advisory boards. The Monitoring and Evaluation unit is probably the largest unit at a Malawian public hospital and provides the opportunity for internal and external research [13, 15]. The research concentrates on topics of HIV-related diseases, prominently TB, antiretroviral treatment and models of care and various groups, e.g. women, children and adolescents (see Table 3). The first publication connected to the Lighthouse appeared in 2006, and this was followed by a significant increase in publications through 2015.

**Table 3:** Published Research involving Lighthouse 2000–2015

	Number of peer reviewed publications	Lighthouse affiliated authors	Lighthouse as study site	Lighthouse data contribution
Operations research/models of care	32	31/32	18/32	14/32
Epidemiological studies	9	9/ 9	6/ 9	3/10
Medical research	11	7/11	4/11	7/11
Other research	1	1/ 1	1/ 1	-
Epidemiological research consortia	41	20/41	na	41/41
Total	102	74	32	70

Papers were classified by main area (by authors NG, FN); see Additional file 2

The Lighthouse as part of the International Epidemiologic Databases to Evaluate AIDS (IeDEA), an international research consortium (http://www.iedea-sa.org/), contributes its cohort data to numerous epidemiological studies.

## Discussion

Since the Lighthouse’s inception in 1997, it has grown from a small group of volunteers to a fully institutionalized organisation with approximately 300 employees. Lighthouse’s portfolio of services has expanded from home-based care for HTC to a fully functional HIV clinical and training centre. A number of strategies and innovations have been integrated into the national programme and become national policy, for example, the nurse-based ART provision and integrated HIV and TB treatment. The introduction of a touch screen-based electronic medical record system was also a breakthrough intervention and has now been installed in over 70 clinics in Malawi [15]. Other interventions, although successful and effective, have not been scaled up into the national programme due to cost, e.g. the Back-to-Care programme.

The Lighthouse’s aforementioned growth was possible because of a massive increase in global funding. HIV and the consequences were of such global importance that an extraordinary amount of funds, through novel funding mechanisms like the Global Fund and PEPFAR, have gone directly into the fight against HIV and AIDS and were further channelled into the national Malawian AIDS response. During the first critical phase of the Lighthouse’s scale up, it represented a structure and organisation that was ready to address HIV and AIDS in an overall health care system in Malawi—a country that was and is facing tremendous challenges. Today, Lighthouse’s operations are largely enabled, but also dependent on its status as local implementing partner of CDC.

Another factor for the Lighthouse’s success was the highly motivated and skilled people who manage and develop the young and growing organisation. Their motivation has inspired additional staff members who have been recruited over the years, which has led to a significant identification with the institution and a sense of ownership. This is also highly related to community links with volunteers and organisations comprised of PLHV. Strong institutional and long-standing partnerships represent a further element of the Lighthouse’s success. Despite receiving significant support, the Lighthouse has managed to be recognized as a strong self-conscious negotiating partner. In return, the Lighthouse has continued to demonstrate the capacity to deliver initiatives of quality management of services and accountability that are all recognized elements of a well-functioning public institution [25].

Since 2005, Lighthouse has developed a recognised and sound profile as a training and research institution. Lighthouse’s future development offers a number of opportunities, but the Lighthouse has to be embedded in the existing landscape of institutions in Malawi, such as the College of Medicine or College of Nursing to keep its unique profile. In regard to research, more Lighthouse staff should assume the role of principal investigator and lead author for scientific publications.

There are a couple of challenges and risks as well as opportunities for institutions like the Lighthouse and their role in ending AIDS.

A decade after the massive scale up HIV and AIDS efforts, Malawi has reached a significant access to ART coverage. In the third quarter of 2015, the HIV programme reported an estimated coverage of people in need of ART as 50% (50,533 / 101,000) for children (<15 years) and 68% (531,990 /779,000) for adults [26]. Aiming to reach the 90–90-90 goals [27] will require addressing harder-to-reach populations and thus the Lighthouse can play a role in developing appropriate strategies. The needs for people with HIV receiving treatment change with increasing age and other health needs such as co-morbidities with non-communicable diseases have to be addressed. The Lighthouse has already started to integrate the screening and treatment of hypertension in its services. However, how much of this care and services should be integrated in an institution like the Lighthouse (originally focused exclusively on HIV) remains to be answered [28].

Some of the Lighthouse’s risks and challenges relate to development of the institution and the Malawian health system. Since the Lighthouse is not an integral part of public health service - as an institution- this provides the Lighthouse with significant managerial freedom and flexibility. However, this may also limit government support when it is needed. Ultimately, the Lighthouse’s model can be seen as in line with the concepts of hospital autonomy as launched by the GoM demonstrating the potential of such an approach [29].

In Malawi, hospitals run by faith-based organisations are often members of a group /association like CHAM (Christian Health Association of Malawi) with 175 member facilities and are backed by the respective churches giving them a stronger negotiation position [30]. However, in contrast, Lighthouse stands on its own and thus requires strong foundations and allied partners.

Lighthouse’s government support is mainly through real estate/land, supplies and support to some staff. Lighthouse depends entirely on external funding and the trust does not generate income by service or training provision. For health care provision, service is free of charge at the point of delivery by governmental policy and an important issue is to offer services to the large number of Malawians living in poverty. Funding by external agencies largely follows a donor-driven agenda with a focus defined by the agency’s portfolio and mandate. Donor country interests can change and make the financial support volatile despite the recipient’s continuing need.

Lighthouse needs to develop a strategy of funding diversification, while at the same time, keeping their high standards to attract external funding.

### Strength and limitations

One strength of this case study is that the reported data include figures from the Lighthouse’s annual reports that are subject to scrutiny by the MoH’s HIV programme, by funding agencies and auditors. The Lighthouse independently commissioned the institutional review, and the OPA review was conducted by a short-term visiting scientist, hence two of the major documents were written by external consultants. Triangulation of the data was possible by the inclusion of interviews with external stakeholders, and participant observation. The long period of inquiry also contributes to the strength of this case study.

We acknowledge that since some authors of this case study are Lighthouse members/employees and other authors have worked as Lighthouse advisors in the past, their judgement of the organization may be biased.

This case study is limited to the Lighthouse as an institution. The wider political and social context is not analysed in depth. Therefore, any conclusions beyond Lighthouse as an institution must be drawn with caution. Nevertheless, we propose that this case study contributes to the discussion of the role of novel institutions such as the Lighthouse in the Malawian health care system and similar settings in other low-income countries.

## Conclusion

Novel institutions like the Lighthouse and other HIV -directed initiatives, organisations and institutions have been developed in low-and middle- income countries around the world. These institutions have demonstrated their capacity to deliver health services and contributed considerably to the current level of success in addressing the disease. However, the position of these sorts of institutions in local health care systems is still being determined.

Lighthouse will need to continue to work on well-planned strategies considering the changing landscape of health needs, provision and financing in Malawi. The Lighthouse needs to find a balance in growth and consolidation, expansion of services and focus on quality health care delivery. In doing so, institutions like the Lighthouse can contribute to the development of health systems in countries like Malawi that improve health care responsiveness and quality for the entire population.

## Additional files

Additional file 1: Interview Topic Guide. The file documents the guiding questions to Lighthouse staff, Ministry of Health employees and external stakeholders. (ODT 8 kb)

Additional file 2: List of Lighthouse publications. This file presents all publications included in Table 3 manuscript and consented to the publication. (DOCX 25 kb)

## Abbreviations

AIDS: Acquired immunodeficiency syndrome
ART: Antiretroviral treatment
BSc: Bachelor of Science
CAFOD: Catholic AID for Overseas Development
CDC: Centers for Disease Control and Prevention
CHAM: Christian Hospital Organization of Malawi
CHBC: Community home based care
CIM: Centre for International Migration and Development ESTHER Ensemble pour une solidarité thérapeutique hospitalière en reseau
GIZ: Deutsche Gesellschaft für Internationale Zusammenarbeit
GoM: Government of Malawi
HIV: Human Immunodeficiency Virus
HTC: HIV testing and counselling centre
IeDEA: International Epidemiologic Databases to Evaluate AIDS
KCH: Kamuzu Central Hospital
LH: Lighthouse
M&E: Monitoring and Evaluation
MoH: Ministry of Health
NGO: Non-governmental organization
NORAD: Norwegian Agency for Development Cooperation
OI: Opportunistic infections
PEPFAR: The U.S. President’s Emergency Plan for AIDS Relief
PhD: Doctor of Philosophy
PLHIV: People living with HIV
SSA: Sub-Saharan-Africa
TASO: The AIDS Support Organisation
UNC: University of North Carolina
VCT/HTC: Voluntary Counselling and Testing /HIV Testing and Counselling
TB: Tuberculosis

## References

[1] UNAIDS. AIDSinfo | UNAIDS. http://aidsinfo.unaids.org/#. Accessed 12 Nov 2015.

[2] Kwanjana JH, Harries AD, Gausi F, Nyangulu DS, Salaniponi FM (2001). TB-HIV seroprevalence in patients with tuberculosis in Malawi. Malawi Med J.

[3] UNAIDS. World AIDS Day 2014 Report: Fact Sheet. 2014.

[4] Matovu s. The AIDS Support Organisation (TASO). http://www.tasouganda.org/. Accessed 6 Aug 2015.

[5] UNAIDS (2014). Malawi: epidemiological fact sheet.

[6] UNICEF. Statistics. 27.12.2013. http://www.unicef.org/infobycountry/malawi_statistics.html. Accessed 6 Aug 2015.

[7] Government of Malawi. Malawi AIDS Response Progress Report: 2015.

[8] World Health Organization Regional Office for Africa. WHO country cooperation strategy. Malawi. 2008-2013:14.07.2009.

[9] World Health Organization. Global Health Observatory Data Repository. http://apps.who.int/gho/data/node.country.country-MWI. Accessed 29 Sept 2015.

[10] World Health Organization. Global Health Observatory Data Repository: Malawi statistics summary (2002 - present).

[11] Stake RE, Denzin NK, Lincoln YS (2005). Qualitative case studies. The SAGE handbook of qualitative research.

[12] Phiri S (2004). The lighthouse: a centre for comprehensive HIV/AIDS treatment and care in Malawi: case study.

[13] Salephera Consulting Ltd. Lighthouse: institutional review report. 2010.

[14] DFID Tuberculosis Knowledge Programme; Tuberculosis related research ProTEST 2001-2005. https://assets.publishing.service.gov.uk/media/57a08c76e5274a27b2001205/HTBLondonProTEST.pdf. Accessed Jan 2016.

[15] Gass T. Innovation, operational research and scaling-up in HIV care: lessons learnt from the lighthouse. Malawi: Organisational and policy analysis (OPA): report; 2011.

[16] Lighthouse Management. Lighthouse financial and administration regulation manual; September 2011.

[17] Kwalamasa K, Bongololo-Mbera G, Namakhoma I, Banda Hastings, Tweya H, Phiri S, et al. Formative research to inform clinic efficienc: qualitative assessment of patient opinions and perceptions of lighthouse trust clinic services. 2012.

[18] Neuhann HF, Hosseinipour MC, Phiri, S. A checklist approach for the continuation of ARV prescriptions in Malawi: 13th ICASA September 2003. Nairobi.

[19] Ministry of Health M, National AIDS Commission Malawi. Treatment of AIDS: Guidelines for the Use of Antiretroviral Therapy in Malawi: National AIDS Commission Malawi; 2008.

[20] Tweya H, Gareta D,Chagwera F, Ben-Smith A, Akua M, Mwenyemasi J, Chiputula F, Boxshall M, Weigel R, Jahn A, Hosseinipour M, Phiri S. Early active follow-up of patients on antiretroviral therapy (ART) who are lost to follow-up: the ‘Back-to-Care’ project in Lilongwe, Malawi. TMIH. 2010;15:(s1)82–9. doi:10.1111/j.1365-3156.2010.02509.x. 10.1111/j.1365-3156.2010.02509.x 20586965

[21] Weigel R, Makwiza I, Nyirenda J, Chiunguzeni D, Phiri S, Theobald S (2009). Supporting children to adhere to anti-retroviral therapy in urban Malawi: multi method insights. BMC Pediatr.

[22] Phiri S, Khan PY, Grant AD, Gareta D, Tweya H, Kalulu M (2011). Integrated tuberculosis and HIV care in a resource-limited setting: experience from the Martin Preuss centre. Malawi Trop Med Int Health.

[23] Tweya H, Ben-Smith A, Kalulu M, Jahn A, Ng'ambi W, Mkandawire E (2014). Timing of antiretroviral therapy and regimen for HIV-infected patients with tuberculosis: the effect of revised HIV guidelines in Malawi. BMC Public Health.

[24] Healthy Developments. Germany's commitment to health and social protection. On behalf of BMZ. Research articles by German-African university and hospital partnerships. 26.09.2014. http://health.bmz.de/what_we_do/Partnerships-for-global-health/Hospital_partnerships__ESTHER_/Research_articles_by_German-African_university_and_hospital_partnerships/index.html. Accessed 17 Feb 2016.

[25] Fryer KJ, Antony J, Douglas A (2007). Critical success factors of continuous improvement in the public sector. TQM Mag.

[26] Government of Malawi, Ministry of Health. Integrated HIV program report: July–September 2015.

[27] UNAIDS. 900–90-90: an ambitious treatment target to help end the AIDS epidemic 2014.

[28] Rabkin M, El-Sadr WM (2011). Why reinvent the wheel? Leveraging the lessons of HIV scale-up to confront non-communicable diseases. Glob Public Health.

[29] Nyasa Times Reporter. Malawi plans hospital fees, nation health fund: Reforms. Nyasa Times. http://www.nyasatimes.com/malawi-plans-hospital-fees-nation-health-healthfund-reforms/. Accessed 29 Apr 2015.

[30] Christian Health Association of Malawi. http://www.cham.org.mw/.

