# Supplementary material for: The path from a volunteer initiative to an established institution: evaluating 15 years of the development and contribution of the Lighthouse trust to the Malawian HIV response
Source: BMC Health Serv Res. 2017 Aug 9;17:548. doi: 10.1186/s12913-017-2466-y (PMC5551033; doi:10.1186/s12913-017-2466-y)
Supplement: Supplementary file 2 — List of Lighthouse publications. This file presents all publications included in Table 3 manuscript and consented to the publication. (DOCX 25 kb) [file 12913_2017_2466_MOESM2_ESM.docx]

**1 Operations research / Models of Care**

1. Ardura-Garcia C, Feldacker C, Tweya H, Chaweza T, Kalulu M, Phiri S, et al. Implementation and Operational Research: Early Tracing of Children Lost to Follow-Up From Antiretroviral Treatment: True Outcomes and Future Risks. J Acquir Immune Defic Syndr. 2015;70:e160-7. doi:10.1097/QAI.0000000000000772.

2. Braun M, Kabue MM, McCollum ED, Ahmed S, Kim M, Aertker L, et al. Inadequate coordination of maternal and infant HIV services detrimentally affects early infant diagnosis outcomes in Lilongwe, Malawi. J Acquir Immune Defic Syndr. 2011;56:e122-8. doi:10.1097/QAI.0b013e31820a7f2f.

3. Feldacker C, Johnson D, Hosseinipour M, Phiri S, Tweya H. Who starts? Factors associated with starting antiretroviral therapy among eligible patients in two, public HIV clinics in Lilongwe, Malawi. PLoS ONE. 2012;7:e50871. doi:10.1371/journal.pone.0050871.

4. Haac BE, Charles AG, Matoga M, LaCourse SM, Nonsa D, Hosseinipour M. HIV testing and epidemiology in a hospital-based surgical cohort in Malawi. World J Surg. 2013;37:2122–8. doi:10.1007/s00268-013-2096-4.

5. Haddad LB, Feldacker C, Jamieson DJ, Tweya H, Cwiak C, Bryant AG, et al. Medical eligibility, contraceptive choice, and intrauterine device acceptance among HIV-infected women receiving antiretroviral therapy in Lilongwe, Malawi. Int J Gynaecol Obstet. 2014;126:213–6. doi:10.1016/j.ijgo.2014.03.026.

6. Haddad LB, Feldacker C, Jamieson DJ, Tweya H, Cwiak C, Chaweza T, et al. Pregnancy prevention and condom use practices among HIV-infected women on antiretroviral therapy seeking family planning in Lilongwe, Malawi. PLoS ONE. 2015;10:e0121039. doi:10.1371/journal.pone.0121039.

7. Hosseinipour MC, Neuhann FH, Kanyama CC, Namarika DC, Weigel R, Miller W, Phiri SJP. Lessons learned from a paying antiretroviral therapy service in the public health sector at Kamuzu Central Hospital, Malawi: 1-year experience. J Int Assoc Physicians AIDS Care (Chic). 2006;5:103–8. doi:10.1177/1545109706288722.

8. Johnson DC, Feldacker C, Tweya H, Phiri S, Hosseinipour MC. Factors associated with timely initiation of antiretroviral therapy in two HIV clinics in Lilongwe, Malawi. Int J STD AIDS. 2013;24:42–9. doi:10.1177/0956462412472312.

9. LaCourse SM, Chester FM, Matoga M, Munthali C, Nsona D, Haac B, et al. Implementation and Operational Research: Implementation of Routine Counselor-Initiated Opt-Out HIV Testing on the Adult Medical Ward at Kamuzu Central Hospital, Lilongwe, Malawi. J Acquir Immune Defic Syndr. 2015;69:e31-5. doi:10.1097/QAI.0000000000000542.

10. Palchaudhuri S, Tweya H, Hosseinipour M. Assessment of laboratory test utilization for HIV/AIDS care in urban ART clinics of Lilongwe, Malawi. Malawi Med J. 2014;26:42–4.

11. Phiri S, Khan PY, Grant AD, Gareta D, Tweya H, Kalulu M, et al. Integrated tuberculosis and HIV care in a resource-limited setting: experience from the Martin Preuss centre, Malawi. Trop Med Int Health. 2011;16:1397–403. doi:10.1111/j.1365-3156.2011.02848.x.

12. Phiri S, Feldacker C, Chaweza T, Mlundira L, Tweya H, Speight C, et al. Integrating reproductive health services into HIV care: strategies for successful implementation in a low-resource HIV clinic in Lilongwe, Malawi. J Fam Plann Reprod Health Care 2015. doi:10.1136/jfprhc-2013-100816.

13. Rosenberg NE, Kamanga G, Phiri S, Nsona D, Pettifor A, Rutstein SE, et al. Detection of acute HIV infection: a field evaluation of the determine® HIV-1/2 Ag/Ab combo test. J Infect Dis. 2012;205:528–34. doi:10.1093/infdis/jir789.

14. Rosenberg NE, van Lettow M, Tweya H, Kapito-Tembo A, Bourdon CM, Cataldo F, et al. Improving PMTCT uptake and retention services through novel approaches in peer-based family-supported care in the clinic and community: a 3-arm cluster randomized trial (PURE Malawi). J Acquir Immune Defic Syndr. 2014;67 Suppl 2:S114-9. doi:10.1097/QAI.0000000000000319.

15. Rutstein SE, Pettifor AE, Phiri S, Kamanga G, Hoffman IF, Hosseinipour MC, et al. Incorporating acute HIV screening into routine HIV testing at sexually transmitted infection clinics and HIV testing and counseling centers in Lilongwe, Malawi. J Acquir Immune Defic Syndr 2015. doi:10.1097/QAI.0000000000000853.

16. Tenthani L, Haas AD, Tweya H, Jahn A, van Oosterhout JJ, Chimbwandira F, et al. Retention in care under universal antiretroviral therapy for HIV-infected pregnant and breastfeeding women (‘Option B+’) in Malawi. AIDS. 2014;28:589–98. doi:10.1097/QAD.0000000000000143.

17. Tweya H, Feldacker C, Ben-Smith A, Weigel R, Boxshall M, Phiri S, Jahn A. 'Task shifting' in an antiretroviral clinic in Malawi: can health surveillance assistants manage patients safely? Public Health Action. 2012;2:178–80. doi:10.5588/pha.12.0018.

18. Tweya H, Ben-Smith A, Kalulu M, Jahn A, Ng'ambi W, Mkandawire E, et al. Timing of antiretroviral therapy and regimen for HIV-infected patients with tuberculosis: the effect of revised HIV guidelines in Malawi. BMC Public Health. 2014;14:183. doi:10.1186/1471-2458-14-183.

19. Tweya H, Feldacker C, Ben-Smith A, Harries AD, Komatsu R, Jahn A, et al. Simplifying ART cohort monitoring: can pharmacy stocks provide accurate estimates of patients retained on antiretroviral therapy in Malawi? BMC Health Serv Res. 2012;12:210. doi:10.1186/1472-6963-12-210.

20. Tweya H, Feldacker C, Phiri S, Ben-Smith A, Fenner L, Jahn A, et al. Comparison of treatment outcomes of new smear-positive pulmonary tuberculosis patients by HIV and antiretroviral status in a TB/HIV clinic, Malawi. PLoS ONE. 2013;8:e56248. doi:10.1371/journal.pone.0056248.

21. Tweya H, Gareta D, Chagwera F, Ben-Smith A, Mwenyemasi J, Chiputula F, et al. Early active follow-up of patients on antiretroviral therapy (ART) who are lost to follow-up: the 'Back-to-Care' project in Lilongwe, Malawi. Trop Med Int Health. 2010;15 Suppl 1:82–9. doi:10.1111/j.1365-3156.2010.02509.x.

22. Tweya H, Gugsa S, Hosseinipour M, Speight C, Ng'ambi W, Bokosi M, et al. Understanding factors, outcomes and reasons for loss to follow-up among women in Option B+ PMTCT programme in Lilongwe, Malawi. Trop Med Int Health. 2014;19:1360–6. doi:10.1111/tmi.12369.

23. Tweya H, Kanyerere H, Ben-Smith A, Kwanjana J, Jahn A, Feldacker C, et al. Re-treatment tuberculosis cases categorised as \"other\": are they properly managed? PLoS ONE. 2011;6:e28034. doi:10.1371/journal.pone.0028034.

24. van Lettow M, Chan AK, Ginsburg AS, Tweya H, Gareta D, Njala J, et al. Timing and uptake of ART during treatment for active tuberculosis in HIV co-infected adults in Malawi. Public Health Action. 2011;1:6–9. doi:10.5588/pha.11.0003.

25. van Oosterhout, Joep J G, Brown L, Weigel R, Kumwenda JJ, Mzinganjira D, Saukila N, et al. Diagnosis of antiretroviral therapy failure in Malawi: poor performance of clinical and immunological WHO criteria. Trop Med Int Health. 2009;14:856–61. doi:10.1111/j.1365-3156.2009.02309.x.

26. Vorkas CK, Tweya H, Mzinganjira D, Dickie G, Weigel R, Phiri S, Hosseinipour MC. Practices to improve identification of adult antiretroviral therapy failure at the Lighthouse Trust clinic in Lilongwe, Malawi. Trop Med Int Health. 2012;17:169–76. doi:10.1111/j.1365-3156.2011.02912.x.

27. Weigel R, Feldacker C, Tweya H, Gondwe C, Chiwoko J, Gumulira J, et al. Managing HIV-infected children in a low-resource, public clinic: a comparison of nurse vs. clinical officer practices in ART refill, calculation of adherence and subsequent appointments. J Int AIDS Soc 2012. doi:10.7448/IAS.15.2.17432.

28. Weigel R, Hochgesang M, Brinkhof MW, Hosseinipour MC, Boxshall M, Mhango E, et al. Outcomes and associated risk factors of patients traced after being lost to follow-up from antiretroviral treatment in Lilongwe, Malawi. BMC Infect Dis. 2011;11:31. doi:10.1186/1471-2334-11-31.

29. Weigel R, Hosseinipour MC, Feldacker C, Gareta D, Tweya H, Chiwoko J, et al. Ensuring HIV-infected pregnant women start antiretroviral treatment: an operational cohort study from Lilongwe, Malawi. Trop Med Int Health. 2012;17:751–9. doi:10.1111/j.1365-3156.2012.02980.x.

30. Weigel R, Kamthunzi P, Mwansambo C, Phiri S, Kazembe PN. Effect of provider-initiated testing and counselling and integration of ART services on access to HIV diagnosis and treatment for children in Lilongwe, Malawi: a pre- post comparison. BMC Pediatr. 2009;9:80. doi:10.1186/1471-2431-9-80.

31. Weigel R, Makwiza I, Nyirenda J, Chiunguzeni D, Phiri S, Theobald S. Supporting children to adhere to anti-retroviral therapy in urban Malawi: multi method insights. BMC Pediatr. 2009;9:45. doi:10.1186/1471-2431-9-45.

32. Weigel R, Phiri S, Chiputula F, Gumulira J, Brinkhof M, Gsponer T, et al. Growth response to antiretroviral treatment in HIV-infected children: a cohort study from Lilongwe, Malawi. Trop Med Int Health. 2010;15:934–44. doi:10.1111/j.1365-3156.2010.02561.x.

**2 Epidemiological Studies**

33. Barnett B, Gokhale RH, Krysiak R, Kanyemba C, Chikaonda T, Bokosi M, et al. Prevalence of drug resistant TB among outpatients at an HIV/TB clinic in Lilongwe, Malawi. Trans R Soc Trop Med Hyg. 2015;109:763–8. doi:10.1093/trstmh/trv092.

34. Glaser N, Deckert A, Phiri S, Rothenbacher D, Neuhann F. Comparison of Various Equations for Estimating GFR in Malawi: How to Determine Renal Function in Resource Limited Settings? PLoS ONE. 2015;10:e0130453. doi:10.1371/journal.pone.0130453.

35. Haddad LB, Cwiak C, Jamieson DJ, Feldacker C, Tweya H, Hosseinipour M, et al. Contraceptive adherence among HIV-infected women in Malawi: a randomized controlled trial of the copper intrauterine device and depot medroxyprogesterone acetate. Contraception. 2013;88:737–43. doi:10.1016/j.contraception.2013.08.006.

36. Hosseinipour MC, van Oosterhout, Joep J G, Weigel R, Phiri S, Kamwendo D, Parkin N, et al. The public health approach to identify antiretroviral therapy failure: high-level nucleoside reverse transcriptase inhibitor resistance among Malawians failing first-line antiretroviral therapy. AIDS. 2009;23:1127–34. doi:10.1097/QAD.0b013e32832ac34e.

37. Speight C, Gabriel L, Phiri S, Tweya H, Sutherland R. Estimation of the true incidence of lactic acidosis within the Lighthouse Clinic cohort, and the likely magnitude of missed diagnoses in the region. J Int AIDS Soc. 2014;17:19558.

38. Tweya H, Feldacker C, Breeze E, Jahn A, Haddad LB, Ben-Smith A, et al. Incidence of pregnancy among women accessing antiretroviral therapy in urban Malawi: a retrospective cohort study. AIDS Behav. 2013;17:471–8. doi:10.1007/s10461-012-0150-0.

39. Tweya H, Feldacker C, Estill J, Jahn A, Ng'ambi W, Ben-Smith A, et al. Are they really lost? \"true\" status and reasons for treatment discontinuation among HIV infected patients on antiretroviral therapy considered lost to follow up in Urban Malawi. PLoS ONE. 2013;8:e75761. doi:10.1371/journal.pone.0075761.

40. Wadonda-Kabondo N, Bennett D, van Oosterhout JJ, Moyo K, Hosseinipour M, Devos J, et al. Prevalence of HIV drug resistance before and 1 year after treatment initiation in 4 sites in the Malawi antiretroviral treatment program. Clin Infect Dis. 2012;54 Suppl 4:S362-8. doi:10.1093/cid/cir987.

41. Wadonda-Kabondo N, Hedt BL, van Oosterhout JJ, Moyo K, Limbambala E, Bello G, et al. A retrospective survey of HIV drug resistance among patients 1 year after initiation of antiretroviral therapy at 4 clinics in Malawi. Clin Infect Dis. 2012;54 Suppl 4:S355-61. doi:10.1093/cid/cis004.

**3 Medical Research**

42. Beadles WI, Jahn A, Weigel R, Clutterbuck D. Peripheral neuropathy in HIV-positive patients at an antiretroviral clinic in Lilongwe, Malawi. Trop Doct. 2009;39:78–80. doi:10.1258/td.2008.080213.

43. Brown KC, Hosseinipour MC, Hoskins JM, Thirumaran RK, Tien H-C, Weigel R, et al. Exploration of CYP450 and drug transporter genotypes and correlations with nevirapine exposure in Malawians. Pharmacogenomics. 2012;13:113–21. doi:10.2217/pgs.11.132.

44. Corbett AH, Hosseinipour MC, Nyirenda J, Kanyama C, Rezk NL, Mkupani P, et al. Pharmacokinetics of generic and trade formulations of lamivudine, stavudine and nevirapine in HIV-infected Malawian children. Antivir Ther (Lond ). 2010;15:83–90. doi:10.3851/IMP1488.

45. Hosseinipour MC, Corbett AH, Kanyama C, Mshali I, Phakati S, Rezk NL, et al. Pharmacokinetic comparison of generic and trade formulations of lamivudine, stavudine and nevirapine in HIV-infected Malawian adults. AIDS. 2007;21:59–64. doi:10.1097/QAD.0b013e3280117ca0.

46. Hosseinipour MC, Sweet KM, Xiong J, Namarika D, Mwafongo A, Nyirenda M, et al. Viral profiling identifies multiple subtypes of Kaposi's sarcoma. MBio. 2014;5:14. doi:10.1128/mBio.01633-14.

47. Jackson AT, Nussbaum JC, Phulusa J, Namarika D, Chikasema M, Kanyemba C, et al. A phase II randomized controlled trial adding oral flucytosine to high-dose fluconazole, with short-course amphotericin B, for cryptococcal meningitis. AIDS. 2012;26:1363–70. doi:10.1097/QAD.0b013e328354b419.

48. Luebbert J, Tweya H, Phiri S, Chaweza T, Mwafilaso J, Hosseinipour MC, et al. Virological failure and drug resistance in patients on antiretroviral therapy after treatment interruption in Lilongwe, Malawi. Clin Infect Dis. 2012;55:441–8. doi:10.1093/cid/cis438.

49. Mwafongo AA, Rosenberg NE, Ng'ambi W, Werner AB, Garneau WM, Gumulira J, et al. Treatment outcomes of AIDS-associated Kaposi's sarcoma under a routine antiretroviral therapy program in Lilongwe, Malawi: bleomycin/vincristine compared to vincristine monotherapy. PLoS ONE. 2014;9:e91020. doi:10.1371/journal.pone.0091020.

50. Nussbaum JC, Jackson A, Namarika D, Phulusa J, Kenala J, Kanyemba C, et al. Combination flucytosine and high-dose fluconazole compared with fluconazole monotherapy for the treatment of cryptococcal meningitis: a randomized trial in Malawi. Clin Infect Dis. 2010;50:338–44. doi:10.1086/649861.

51. Phiri S, Hoffman IF, Weiss HA, Martinson F, Nyirenda N, Kamwendo D, et al. Impact of aciclovir on ulcer healing, lesional, genital and plasma HIV-1 RNA among patients with genital ulcer disease in Malawi. Sex Transm Infect. 2010;86:345–52. doi:10.1136/sti.2009.041814.

52. Phiri S, Zadrozny S, Weiss HA, Martinson F, Nyirenda N, Chen C-Y, et al. Etiology of genital ulcer disease and association with HIV infection in Malawi. Sex Transm Dis. 2013;40:923–8. doi:10.1097/OLQ.0000000000000051.

**4) Other Research**

53. Tabatabai J, Namakhoma I, Tweya H, Phiri S, Schnitzler P, Neuhann F. Understanding reasons for treatment interruption amongst patients on antiretroviral therapy--a qualitative study at the Lighthouse Clinic, Lilongwe, Malawi. Glob Health Action. 2014;7:24795.

**5 Epidemiological Research Consortia**

54. A biregional survey and review of first-line treatment failure and second-line paediatric antiretroviral access and use in Asia and southern Africa. J Int AIDS Soc. 2011;14:7. doi:10.1186/1758-2652-14-7.

55. A survey of paediatric HIV programmatic and clinical management practices in Asia and sub-Saharan Africa--the International epidemiologic Databases to Evaluate AIDS (IeDEA). J Int AIDS Soc. 2013;16:17998. doi:10.7448/IAS.16.1.17998.

56. Arrivé E, Kyabayinze DJ, Marquis B, Tumwesigye N, Kieffer M-P, Azondekon A, et al. Cohort profile: the paediatric antiretroviral treatment programmes in lower-income countries (KIDS-ART-LINC) collaboration. Int J Epidemiol. 2008;37:474–80. doi:10.1093/ije/dym216.

57. Avila D, Althoff KN, Mugglin C, Wools-Kaloustian K, Koller M, Dabis F, et al. Immunodeficiency at the start of combination antiretroviral therapy in low-, middle-, and high-income countries. J Acquir Immune Defic Syndr. 2014;65:e8-16. doi:10.1097/QAI.0b013e3182a39979.

58. Ballif M, Nhandu V, Wood R, Dusingize JC, Carter EJ, Cortes CP, et al. Detection and management of drug-resistant tuberculosis in HIV-infected patients in lower-income countries. Int J Tuberc Lung Dis. 2014;18:1327–36. doi:10.5588/ijtld.14.0106.

59. Ballif M, Renner L, Claude Dusingize J, Leroy V, Ayaya S, Wools-Kaloustian K, et al. Tuberculosis in Pediatric Antiretroviral Therapy Programs in Low- and Middle-Income Countries: Diagnosis and Screening Practices. J Pediatric Infect Dis Soc. 2015;4:30–8. doi:10.1093/jpids/piu020.

60. Braitstein P, Boulle A, Nash D, Brinkhof MWG, Dabis F, Laurent C, et al. Gender and the use of antiretroviral treatment in resource-constrained settings: findings from a multicenter collaboration. J Womens Health (Larchmt). 2008;17:47–55. doi:10.1089/jwh.2007.0353.

61. Brinkhof MWG, Boulle A, Weigel R, Messou E, Mathers C, Orrell C, et al. Mortality of HIV-infected patients starting antiretroviral therapy in sub-Saharan Africa: comparison with HIV-unrelated mortality. PLoS Med. 2009;6:e1000066. doi:10.1371/journal.pmed.1000066.

62. Brinkhof MWG, Dabis F, Myer L, Bangsberg DR, Boulle A, Nash D, et al. Early loss of HIV-infected patients on potent antiretroviral therapy programmes in lower-income countries. Bull World Health Organ. 2008;86:559–67.

63. Brinkhof MWG, Egger M, Boulle A, May M, Hosseinipour M, Sprinz E, et al. Tuberculosis after initiation of antiretroviral therapy in low-income and high-income countries. Clin Infect Dis. 2007;45:1518–21. doi:10.1086/522986.

64. Brinkhof MWG, Spycher BD, Yiannoutsos C, Weigel R, Wood R, Messou E, et al. Adjusting mortality for loss to follow-up: analysis of five ART programmes in sub-Saharan Africa. PLoS ONE. 2010;5:e14149. doi:10.1371/journal.pone.0014149.

65. Calmy A, Balestre E, Bonnet F, Boulle A, Sprinz E, Wood R, et al. Mean CD4 cell count changes in patients failing a first-line antiretroviral therapy in resource-limited settings. BMC Infect Dis. 2012;12:147. doi:10.1186/1471-2334-12-147.

66. Davies M-A, May M, Bolton-Moore C, Chimbetete C, Eley B, Garone D, et al. Prognosis of children with HIV-1 infection starting antiretroviral therapy in Southern Africa: a collaborative analysis of treatment programs. Pediatr Infect Dis J. 2014;33:608–16. doi:10.1097/INF.0000000000000214.

67. Davies M-A, Moultrie H, Eley B, Rabie H, van Cutsem G, Giddy J, et al. Virologic failure and second-line antiretroviral therapy in children in South Africa--the IeDEA Southern Africa collaboration. J Acquir Immune Defic Syndr. 2011;56:270–8. doi:10.1097/QAI.0b013e3182060610.

68. Davies M-A, Phiri S, Wood R, Wellington M, Cox V, Bolton-Moore C, et al. Temporal trends in the characteristics of children at antiretroviral therapy initiation in southern Africa: the IeDEA-SA Collaboration. PLoS ONE. 2013;8:e81037. doi:10.1371/journal.pone.0081037.

69. Duda SN, Farr AM, Lindegren ML, Blevins M, Wester CW, Wools-Kaloustian K, et al. Characteristics and comprehensiveness of adult HIV care and treatment programmes in Asia-Pacific, sub-Saharan Africa and the Americas: results of a site assessment conducted by the International epidemiologic Databases to Evaluate AIDS (IeDEA) Collaboration. J Int AIDS Soc. 2014;17:19045. doi:10.7448/IAS.17.1.19045.

70. Egger M, Spycher BD, Sidle J, Weigel R, Geng EH, Fox MP, et al. Correcting mortality for loss to follow-up: a nomogram applied to antiretroviral treatment programmes in sub-Saharan Africa. PLoS Med. 2011;8:e1000390. doi:10.1371/journal.pmed.1000390.

71. Estill J, Egger M, Johnson LF, Gsponer T, Wandeler G, Davies M-A, et al. Monitoring of antiretroviral therapy and mortality in HIV programmes in Malawi, South Africa and Zambia: mathematical modelling study. PLoS ONE. 2013;8:e57611. doi:10.1371/journal.pone.0057611.

72. Estill J, Tweya H, Egger M, Wandeler G, Feldacker C, Johnson LF, et al. Tracing of patients lost to follow-up and HIV transmission: mathematical modeling study based on 2 large ART programs in Malawi. J Acquir Immune Defic Syndr. 2014;65:86. doi:10.1097/QAI.0000000000000075.

73. Feldacker C, Tweya H, Keiser O, Weigel R, Kalulu M, Fenner L, et al. Characteristics of adults and children diagnosed with tuberculosis in Lilongwe, Malawi: findings from an integrated HIV/TB clinic. Trop Med Int Health. 2012;17:1108–16. doi:10.1111/j.1365-3156.2012.03041.x.

74. Fenner L, Forster M, Boulle A, Phiri S, Braitstein P, Lewden C, et al. Tuberculosis in HIV programmes in lower-income countries: practices and risk factors. Int J Tuberc Lung Dis. 2011;15:620–7. doi:10.5588/ijtld.10.0249.

75. Fenner L, Ballif M, Graber C, Nhandu V, Dusingize JC, Cortes CP, et al. Tuberculosis in antiretroviral treatment programs in lower income countries: availability and use of diagnostics and screening. PLoS ONE. 2013;8:e77697. doi:10.1371/journal.pone.0077697.

76. Gsponer T, Petersen M, Egger M, Phiri S, Maathuis MH, Boulle A, et al. The causal effect of switching to second-line ART in programmes without access to routine viral load monitoring. AIDS. 2012;26:57–65. doi:10.1097/QAD.0b013e32834e1b5f.

77. Gsponer T, Weigel R, Davies M-A, Bolton C, Moultrie H, Vaz P, et al. Variability of growth in children starting antiretroviral treatment in southern Africa. Pediatrics. 2012;130:e966-77. doi:10.1542/peds.2011-3020.

78. Haas AD, Keiser O, Balestre E, Brown S, Bissagnene E, Chimbetete C, et al. Monitoring and switching of first-line antiretroviral therapy in adult treatment cohorts in sub-Saharan Africa: Collaborative analysis. The Lancet HIV. 2015;2:e271-e278. doi:10.1016/S2352-3018(15)00087-9.

79. Hoskins S, Jahn A, Somi G, Semenenko I, Kirungi W, Kaleebu P, et al. Evaluating the systems used to monitor HIV populations accessing therapy and care in low-income and lower-middle-income countries. AIDS. 2012;26 Suppl 2:S137-45. doi:10.1097/QAD.0b013e32835bde24.

80. Huisin 't Veld D, Balestre E, Buyze J, Menten J, Jaquet A, Cooper DA, et al. Determinants of Weight Evolution Among HIV-Positive Patients Initiating Antiretroviral Treatment in Low-Resource Settings. J Acquir Immune Defic Syndr. 2015;70:146–54. doi:10.1097/QAI.0000000000000691.

81. Hutchinson E, Parkhurst J, Phiri S, Di Gibb M, Chishinga N, Droti B, Hoskins S. National policy development for cotrimoxazole prophylaxis in Malawi, Uganda and Zambia: the relationship between Context, Evidence and Links. Health Res Policy Syst. 2011;9 Suppl 1:S6. doi:10.1186/1478-4505-9-S1-S6.

82. Keiser O, Anastos K, Schechter M, Balestre E, Myer L, Boulle A, et al. Antiretroviral therapy in resource-limited settings 1996 to 2006: patient characteristics, treatment regimens and monitoring in sub-Saharan Africa, Asia and Latin America. Trop Med Int Health. 2008;13:870–9. doi:10.1111/j.1365-3156.2008.02078.x.

83. Keiser O, Blaser N, Davies M-A, Wessa P, Eley B, Moultrie H, et al. Growth in Virologically Suppressed HIV-Positive Children on Antiretroviral Therapy: Individual and Population-level References. Pediatr Infect Dis J. 2015;34:e254-9. doi:10.1097/INF.0000000000000801.

84. Keiser O, Chi BH, Gsponer T, Boulle A, Orrell C, Phiri S, et al. Outcomes of antiretroviral treatment in programmes with and without routine viral load monitoring in Southern Africa. AIDS. 2011;25:1761–9. doi:10.1097/QAD.0b013e328349822f.

85. Keiser O, Tweya H, Boulle A, Braitstein P, Schecter M, Brinkhof MWG, et al. Switching to second-line antiretroviral therapy in resource-limited settings: comparison of programmes with and without viral load monitoring. AIDS. 2009;23:1867–74. doi:10.1097/QAD.0b013e32832e05b2.

86. Keiser O, Tweya H, Braitstein P, Dabis F, MacPhail P, Boulle A, et al. Mortality after failure of antiretroviral therapy in sub-Saharan Africa. Trop Med Int Health. 2010;15:251–8. doi:10.1111/j.1365-3156.2009.02445.x.

87. Leroy V, Malateste K, Rabie H, Lumbiganon P, Ayaya S, Dicko F, et al. Outcomes of antiretroviral therapy in children in Asia and Africa: a comparative analysis of the IeDEA pediatric multiregional collaboration. J Acquir Immune Defic Syndr. 2013;62:208–19. doi:10.1097/QAI.0b013e31827b70bf.

88. May M, Boulle A, Phiri S, Messou E, Myer L, Wood R, et al. Prognosis of patients with HIV-1 infection starting antiretroviral therapy in sub-Saharan Africa: A collaborative analysis of scale-up programmes. The Lancet. 2010;376:449–57. doi:10.1016/S0140-6736(10)60666-6.

89. Nash D, Katyal M, Brinkhof MWG, Keiser O, May M, Hughes R, et al. Long-term immunologic response to antiretroviral therapy in low-income countries: a collaborative analysis of prospective studies. AIDS. 2008;22:2291–302. doi:10.1097/QAD.0b013e3283121ca9.

90. Porter M, Davies M-A, Mapani MK, Rabie H, Phiri S, Nuttall J, et al. Outcomes of Infants Starting Antiretroviral Therapy in Southern Africa, 2004–2012. JAIDS Journal of Acquired Immune Deficiency Syndromes. 2015;69:593–601. doi:10.1097/QAI.0000000000000683.

91. Schomaker M, Egger M, Ndirangu J, Phiri S, Moultrie H, Technau K, et al. When to start antiretroviral therapy in children aged 2-5 years: a collaborative causal modelling analysis of cohort studies from southern Africa. PLoS Med. 2013;10:e1001555. doi:10.1371/journal.pmed.1001555.

92. Spaar A, Graber C, Dabis F, Coutsoudis A, Bachmann L, McIntyre J, et al. Prioritising prevention strategies for patients in antiretroviral treatment programmes in resource-limited settings. AIDS Care. 2010;22:775–83. doi:10.1080/09540120903349102.

93. Tassie J-M, Malateste K, Pujades-Rodriguez M, Poulet E, Bennett D, Harries A, et al. Evaluation of three sampling methods to monitor outcomes of antiretroviral treatment programmes in low- and middle-income countries. PLoS ONE. 2010;5:e13899. doi:10.1371/journal.pone.0013899.

94. Tuboi SH, Pacheco AG, Harrison LH, Stone RA, May M, Brinkhof MWG, et al. Mortality associated with discordant responses to antiretroviral therapy in resource-constrained settings. J Acquir Immune Defic Syndr. 2010;53:70–7. doi:10.1097/QAI.0b013e3181c22d19.
