# Supplementary material for: The path from a volunteer initiative to an established institution: evaluating 15 years of the development and contribution of the Lighthouse trust to the Malawian HIV response
Source: BMC Health Serv Res. 2017 Aug 9;17:548. doi: 10.1186/s12913-017-2466-y (PMC5551033; doi:10.1186/s12913-017-2466-y)
Supplement: Supplementary file 3 [file 12913_2017_2466_MOESM1_ESM.odt]

Appendix: Interview Topic Guide

Information Sheet and Informed Consent Form given to all interviewees.

A. Lighthouse staff (clinic, HTC, HBC)

- Your department, function, years of employment with Lighthouse, professional background, age.

- What are your tasks in the delivery of HIV/Aids services at the Lighthouse?

- In your opinion, what are presently the strengths and challenges of your programme / service?

- If you had the means and competence to do so, is there anything you would change or introduce to improve the service / programme?

- How do you share your experiences and opinion on challenges and opportunities internally?

- What has been the most important new component of your programme / service that was introduced during the last two years?

- How are you involved in planning of activities and of the programme?

- Would you call any component of your programme an innovation?

- How do you measure effectiveness and quality of your programme / service?

- How do you utilize M&E reports?

- Are you involved when conculsions are drawn from M&E and OR findings? How are conclusions drawn?

- Do you share experiences, lessons and findings of your programme / service with external organisations or persons? If yes, by what means?

- How are such lessons documented internally?

- In your working day, on what occasions do you communicate experience with your colleagues? What are the formal communication channels? What are informal communication channels?

- Do you have access to external resources and learning about your topic? What kind?

- Do your supervisors expect from you that you contribute to learning, reflection and quality improvement?

- Do you have time and space for learning and reflection?

- How is your collaboration with the Ministry of Health?

- With whom do you work in the MoH?

- Do you have personal contacts to people working in the MoH?

- In your view, what are the strenghts and weaknesses of the MoH concerning your working area?

- Have any components of services / programmes of the LH have been taken over and scaled up by the MoH?

- If yes, how was the process?

B. Ministry of Health

- What are your personal experiences on collaboration with the Lighthouse?

- In your view, what does the LH contribute to the development of the Malawi health sector?

- In your opinion, what has been the most important innovations in HIV/Aids services in Malawi, where LH was involved?

- Why is the LH important for the MoH? How does the MoH benefit of the LH?

- In what areas could the LH improve their performance?

- At the MPC, the LH is hosting the national TB programme? What is your experience with this partnership? What are the challenges?

- Which is the most important project currently running between the MoH and the LH? Future plans?

- What innovations do you plan to scale up in the near future?

C. Other stakeholders

- What are your personal experiences on collaboration with the Lighthouse?

- In your view, what does the LH contribute to the development of the Malawi health sector?

- In your opinion, what has been the most important innovations in HIV/Aids services in Malawi, where LH was involved?

- Why is the LH important for the MoH? How does the MoH benefit of the LH?

- In what areas could the LH improve their performance?

- At the MPC, the LH is hosting the national TB programme? What is your experience with this partnership? What are the challenges?

- Which is the most important project currently running between the MoH and the LH? Future plans?

- What innovations do you plan to scale up in the near future?
